# In-Line Ultrasonic Monitoring for Sediments Stuck on Inner Wall of a Polyvinyl Chloride Pipe

**DOI:** 10.1155/2014/731621

**Published:** 2014-08-28

**Authors:** Hogeon Seo, Kyoungjun Lee, Kyung-Young Jhang

**Affiliations:** ^1^Department of Mechanical Convergence Engineering, Hanyang University, Seoul 133-791, Republic of Korea; ^2^School of Mechanical Engineering, Hanyang University, Seoul 133-791, Republic of Korea

## Abstract

This research verified the applicability and effectiveness of the ultrasonic monitoring of sediments stuck on the inner wall of polyvinyl chloride (PVC) pipes. For identifying the transmittance of acoustic energy and the speed of sound in the PVC material, the pulse-echo ultrasonic testing was conducted for PVC sheets of different thicknesses. To simulate the solidified sediment, the hot melt adhesive (HMA) was covered on the inner wall of the PVC pipe in different heights. From the experiment, the speeds of sound in the PVC and the HMA materials were obtained as about 2258 and 2000 m/s, respectively. The thickness of the materials was calculated through the signal processing such as taking the absolute value and low pass filtering, the echo detection, and the measurement of the time of flight. The errors between actual and measured thicknesses of PVC sheets were below 5%. In the case of the substance stuck on the inner wall, the errors were below 2.5%. Since the pulse-echo ultrasonic inspection is available on the outer surface and its measurement accuracy was over 95%, it can be an efficient and effective in-service structural health monitoring for the sediment on the wall of PVC pipes.

## 1. Introduction

Polyvinyl chloride (PVC) plastic is amorphous and provides good performance at low cost; it has been widely used for a variety of industrial purposes as well as infrastructural and building applications such as fluid and gas distribution networks [1–3]. In particular, in the manufacturing field, various kinds of materials are delivered from huge tanks to each machine through pipe networks. In the case of inviscid flow, internal corrosion and surface cracks are serious issues threating the structural health and safety in PVC pipelines. On the other hand, when viscous or adhesive materials such as paint, cement, gel, and liquid polymer flow in the PVC pipe, they can stick to the inner wall of PVC pipes due to their viscosity and then become solidified. As the sediment sticks thicker on the inner wall, the internal pressure in the pipeline increases. This can result in not only the flow blockage and even its explosion but also huge productivity losses [4, 5]. For this reason, the accumulation of the sediment on the inner wall of PVC pipes has raised one of the critical issues in terms of the structural maintenance and productivity.

For the advanced prevention caused by the sediment deposition on the inner wall of the PVC pipeline, it is necessary to measure the thickness of the stuck material. However, in general, in order to inspect the inner state of the pipeline, the pipes should be disassembled independently of each other and then each pipe can be examined individually. Since this kind of structural maintenance for PVC pipelines requires the suspension of operation, it is not time- and cost-efficient. To save time and money, the in-service nondestructive evaluation (NDE) for the inner part of the pipeline should be introduced without intermission in operation [6]. That technique should be able to diagnose the inside of the pipe at its outer wall without the separation of pipes from the entire pipeline.

Ultrasonic testing (UT) can be one of the useful NDE techniques for the inspection of the pipeline [5]. In UT, the acoustic waves are transmitted into materials and then the material characteristics or properties are evaluated through detecting and analyzing the reflected or transmitted signal [7–9]. Ultrasonic testing has been used for a variety of applications such as detecting surface and subsurface defects and measuring the depth of flaws, the thickness of materials, and the rate of flow because ultrasonic measurements are rapid, accurate, and nondestructive; moreover, it can be fully automated and conducted in-service [4, 6, 9–11]. For the polymeric materials and processes, ultrasounds have been used for numerous purposes such as polymer blending [12–16], characterization of polymers in both solid and liquid states [17–22], the measurements of wave velocities and attenuation [23–28], and the monitoring of the flow in plastic pipes [29–31]. However, the UT application of monitoring for the inner wall of the PVC pipe is rare although PVC pipe has been widely used for many fields. Through utilizing the many advantages of UT, the inside state of the PVC pipes can be monitored in-service. Then, it can be possible to diagnose and prevent the potential threats such as the flow blockage in pipe and its explosion in advance.

As a practical application of UT for manufacturing industry, this research studied the applicability and effectiveness of the ultrasonic monitoring for the sediment stuck on the inner wall of a PVC pipe. Since the inspection should work on the outer surface of PVC pipes and measure the thickness of materials, a pulse-echo ultrasonic measurement was proposed in this research [32]. With PVC sheets and hot melt adhesive (HMA), that is, also known as hot glue gun stick, the transmittance of ultrasonic waves in the PVC material and the speed of sound in the PVC and sediment materials were investigated. The HMA was used to simulate the solidified sediment in order to verify its practical effectiveness.

## 2. Theory

In a certain medium, acoustic waves travel with the speed of sound dependent on stiffness and density of the medium they are passing through. Generally, the speed of sound *c* is given by the Newton-Laplace equation:

(1) $$\begin{array}{c} c=\sqrt{\frac{K}{\rho }}, \end{array}$$

where *K* is a coefficient of stiffness or the bulk modulus and *ρ* is the density. The speed of sound is constant in the uniform material under the same circumstances such as temperature and stress state. Thus, by measuring the time of flight of the acoustic wave, the travel distance can be calculated.

**Figure 1:**
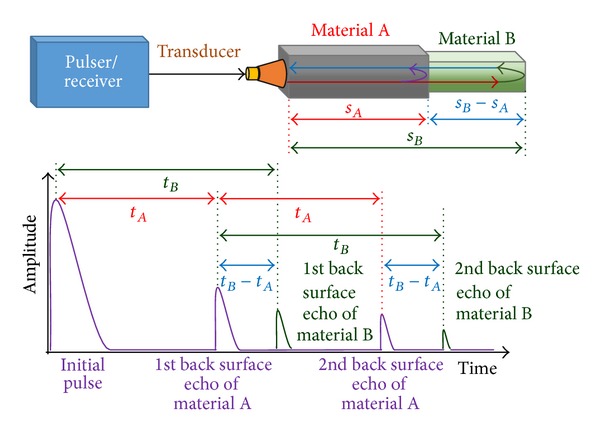
Pulse-echo ultrasonic method for two materials.

Figure 1 shows the principle of the pulse-echo method. A pulser/receiver supplies a high voltage electrical pulse to the transducer and amplifies the received ultrasonic wave signal. In the case of single material, the wave is almost reflected when it meets boundaries that have a significant difference in acoustic impedances (*Z*) of the materials on each side of the boundary, such as a discontinuity or the back wall [33, 34]. On the other hand, in the case of multimaterials that have similar acoustic impedances, some wave energy transmits to the neighboring material and the rest is reflected. The transmitted wave is eventually reflected at the back surface. Even though part of the sound energy is attenuated, reflected signals can be detected if the amplitude of the initial pulse is high enough.

Since the reflected signal travels back and forth between front and back surface, the travel distance and travel time of the wave have the following relationship:

(2) $$\begin{array}{c} t=\frac{2s}{c}, \end{array}$$

where *t* is the time of flight (TOF), *s* is the one-way travel distance or the thickness of the material, and *c* is the speed of sound in the material. Thus, the speed of sound in a certain material can be calculated when we know its thickness. Conversely, the thickness of a material can be calculated by measuring the TOF of acoustic waves when the speed of sound of the material is known. Likewise, the thickness of a neighboring material can be calculated by measuring the TOF in the neighboring material as follows:

(3) $$\begin{array}{c} s_{B-A}=s_{B}-s_{A}=c_{B}\times \frac{t_{B}-t_{A}}{2}, \end{array}$$

where *s*_*B*−*A*_ is the one-way travel distance or the thickness of the material *B*, *t*_*A*_ is the TOF of the first reflected echo from the back surface of material *A*, *t*_*B*_ is the TOF of the first reflected echo from the back surface of material *B*, and *c*_*B*_ is the speed of sound in the material *B*.

While the ultrasonic wave travels through the medium, its amplitude exponentially diminishes with the propagation distance. Although the amplitude reduction is ideally caused by the spreading of the wave, scattering and absorption weaken the ultrasound in natural materials. Scattering is the reflection of the wave in many directions and absorption is the conversion of the sound energy into other forms of energy. The combined effect of them is called attenuation. Ultrasonic attenuation is the decay rate of the ultrasonic wave while it propagates through material. The amplitude variation of a decaying wave can be expressed as

(4) $$\begin{array}{c} A=A_{0}e^{-\alpha x}, \end{array}$$

where *x* is the propagation distance, *A*_0_ is the amplitude at *x*_0_, *A*_*x*_ is the amplitude at *x*, and *α* is the attenuation coefficient. From (4), we can obtain the attenuation coefficient as follows:

(5) $$\begin{array}{c} \alpha =\frac{1}{x}\ln\left(\frac{A_{0}}{A_{x}}\right)\;\left[\text{Np}/\text{m}\right]. \end{array}$$

The units of the attenuation value in neper per meter (Np/m) can be converted to decibels per meter (dB/m) by multiplying by 8.686.

## 3. Specimen and Experiments

**Figure 2:**
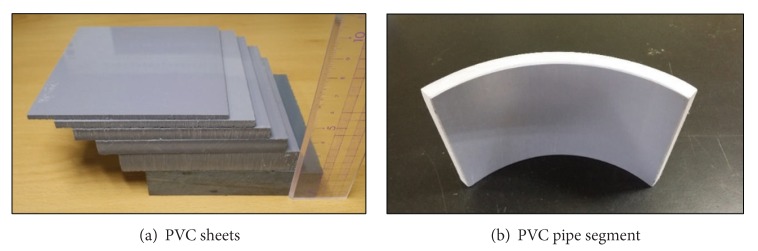
PVC specimens: (a) sheets and (b) pipe segment.

PVC specimens used for the pulse-echo ultrasonic testing are as shown in Figure 2. Each sheet is 100 mm wide and 100 mm tall. The thicknesses of them are 3, 5, 8, 10, 15, and 20 mm. PVC sheets were used for the measurement of the speed of sound, the ultrasonic transmittance, and the attenuation coefficient of sound waves in PVC material. PVC pipe was used to evaluate the effectiveness of the practical application of the pulse-echo ultrasonic inspection on the curved surface to measure the thickness of adhesive substance stuck on the inner wall of the pipe. The nominal size of the pipe is 200 mm and its wall thickness is 10.3 mm.

**Figure 3:**
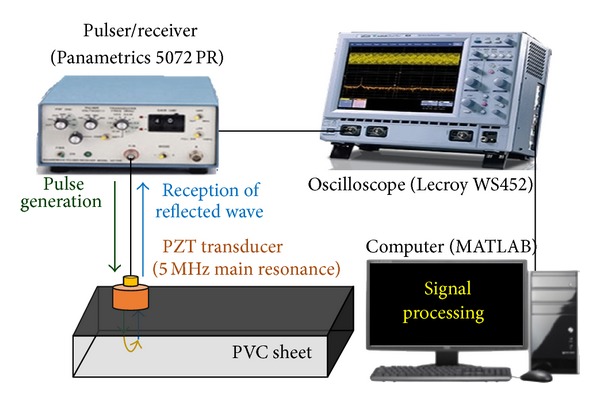
Pulse-echo ultrasonic inspection for PVC sheets.

In order to evaluate the ultrasonic transmittance in PVC materials, TOFs in PVC sheets of different thicknesses were measured in the pulse-echo ultrasonic inspection as shown in Figure 3. When the pulser/receiver excites a negative electrical pulse, the PZT transducer is driven to produce acoustic waves. If the amplitude of the initial pulse is high enough to return to the PZT transducer, the echo signals reflected from the opposite surface are detected. The digital oscilloscope converts the electrical signal into the digital data through the embedded analog/digital converter and displays the transmitted and reflected waves. By signal processing of the digital data in MATLAB software, the speed of sound in the material and the thicknesses of specimens are calculated from (2). The attenuation coefficient of sound waves was additionally obtained.

HMA is used to simulate the adhesive substance on the inner wall of PVC pipe. In the pipe, the critical threat to its safety is not just sticky fluids but the fluid substance that adheres to the wall and becomes solidified. The accumulation of the hard sediment on the inner wall obstructs the flow, which increases the internal pressure in the pipe. This causes flow blockage in pipe and even its explosion. In terms of being solidified from the fluid state, the glue gun stick has similar material characteristics with the sticky sediments on the wall of PVC pipes. When it is hot, it is fluid. As it cools down, it becomes hard and ends up as a solid.

To investigate the effectiveness of the pulse-echo ultrasonic monitoring for sediments on the wall of the PVC pipe, HMA was squeezed onto the inner wall of the PVC pipe. Different thicknesses of solidified substances were formed by controlling the amount of the HMA. The thicknesses of the HMA covered on the wall of the PVC pipe are 3.92, 6.79, 7.49, 10.05, and 11.63 mm.

**Figure 4:**
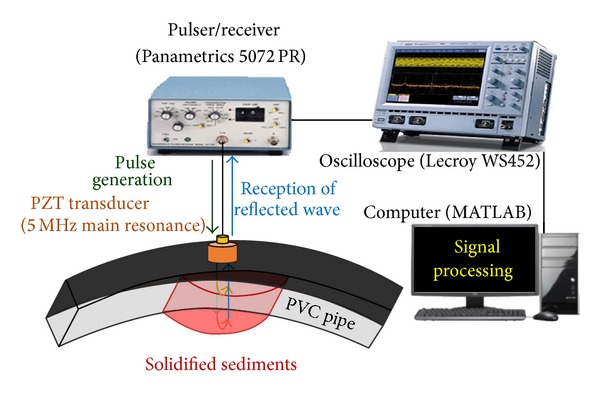
Pulse-echo ultrasonic inspection for PVC pipe.

Likewise with the experiment for PVC sheets, TOFs in PVC pipe and the solidified adhesive of different thicknesses were measured in the pulse-echo ultrasonic inspection as shown in Figure 4. Unlike PVC sheets, some acoustic energy transmits from the PVC pipe into the adhesive material and the rest is reflected at the interface between the PVC pipe and the solidified adhesive. If the amplitude of the initial pulse is high enough to return to the PZT transducer even after transmitting into the solidified adhesive material, the acoustic wave will be reflected from the back surface of the adhesive substance and this echo signal can be detected. Through signal processing in MATLAB, the speed of sound in the HMA and the thicknesses of the adhesive substance on the inner of wall of the PVC pipe are calculated from (3).

## 4. Experimental Results

**Figure 5:**
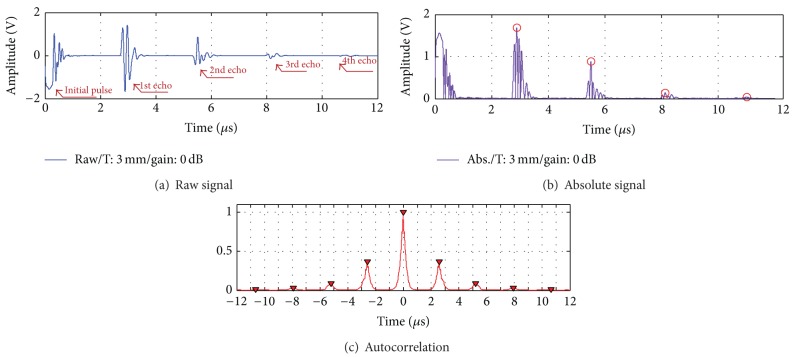
Signals of PVC sheets (T: 3 mm/gain: 0 dB): (a) raw, (b) absolute, and (c) autocorrelation.

From the experiment for the PVC sheets, the speed of sound and the attenuation coefficient in PVC material were measured. With the PVC sheet of 3 mm thickness, the pulse-echo signals were detected by PZT transducer as shown in Figure 5(a). In order to calculate the speed of sound in the PVC material, TOFs between pulse-echoes should be obtained. In measuring the thickness of specimens and the speed of sound by obtaining TOFs, the accuracy of time measurement is very significant. Since there can be phase conversion of the pulse wave at the boundary or ringing can occur, selecting proper peaks is critical to exactly calculate TOFs. To make this easy and accurate, several digital signal processing techniques are necessary.

First, to the speed of sound in the PVC material, the absolute value of the raw signal was taken as shown in Figure 5(b). Since the autocorrelation method is effective to detect repeats or periodicity in a signal, the autocorrelation processing was used in order to more accurately measure the TOFs between pulse-echoes as shown in Figure 5(c). The measured TOFs are as shown in Table 1. From these results and thickness of the PVC sheet, the calculated speed of sound was calculated as about 2258 m/s, that is, similar as the well-known speed of sound in the PVC material.

**Figure 6:**
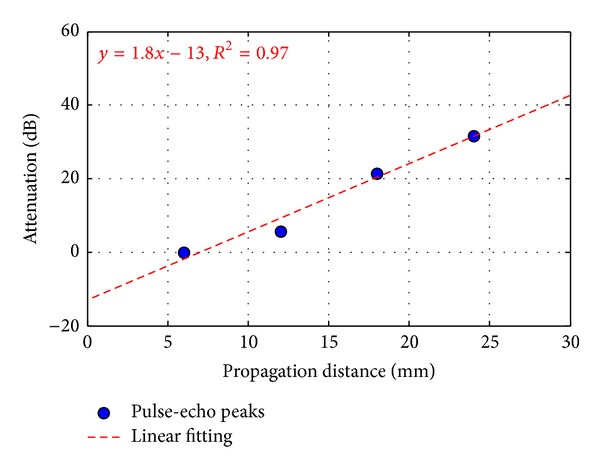
Attenuation depending on propagation distance.

After the pulse-echo peaks were detected as shown in Figure 5(b), the attenuation coefficient in the PVC material was calculated by (5).  Figure 6 shows the attenuation of acoustic energy in the PVC sheet of 3 mm thickness. By linear fitting of attenuation, the attenuation coefficient in the PVC material (*α*_PVC_) was obtained as about 1.8 dB/mm.

**Figure 7:**
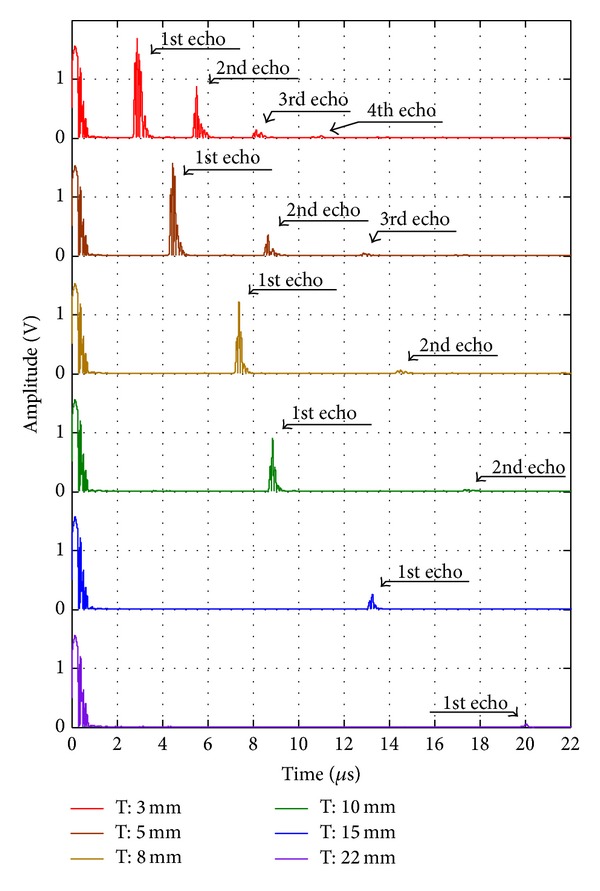
Reflected waves in PVC sheets of different thickness (gain: 0 dB).

Ultrasonic transmittance in PVC material was investigated through the reflected signal measurement for the PVC sheets of different thicknesses. In order to detect pulse-echo signals clearly, the absolute values of the raw signals were taken as shown in Figure 7. Each signal measured from the PVC sheets of different thicknesses is as shown in different colors. As the thickness of the PVC sheet became thicker, the TOF increased proportionally as shown in Table 2. The measured thickness of the PVC sheets was calculated from (2). The errors between actual and measured thicknesses were below 5%, which means that an ultrasonic pulse is able to propagate deeper than 40 mm without the significant frequency variation.

**Figure 8:**
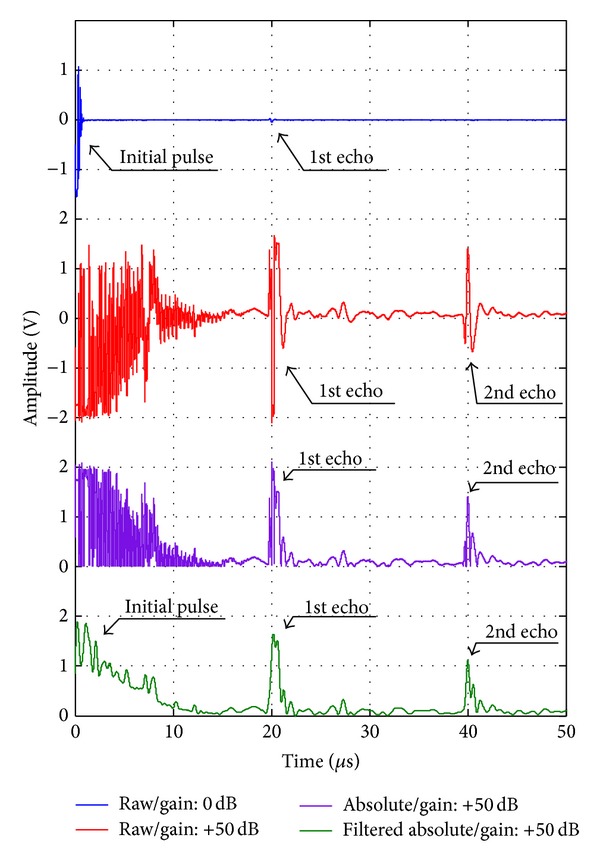
Signal processing of the reflected ultrasonic wave: taking absolute value of the raw signal and the filtered-out signal of the absolute value.

However, as the propagation depth was deeper, the amplitude decreased exponentially as shown in Figure 7. For amplifying the low amplitude signal, the gain of pulser/receiver was increased up to +50 dB and the pulse-echo ultrasonic measurement was conducted for the PVC sheet of 22 mm thickness. The experimental results and their signal processing results are as shown in Figure 8. In the gain of 0 dB, the reflected signal was detected but was too small compared to the initial pulse, whereas the first and second echo signals were obvious in the gain of +50 dB. In order to detect echo-pulses more clearly, the absolute value of the received signal was taken, and then the high frequency components were filtered out with a low pass filter. In consequence of that signal processing, the pulse-echo detection from the processed data is easier and clearer than from the raw signal.

**Figure 9:**
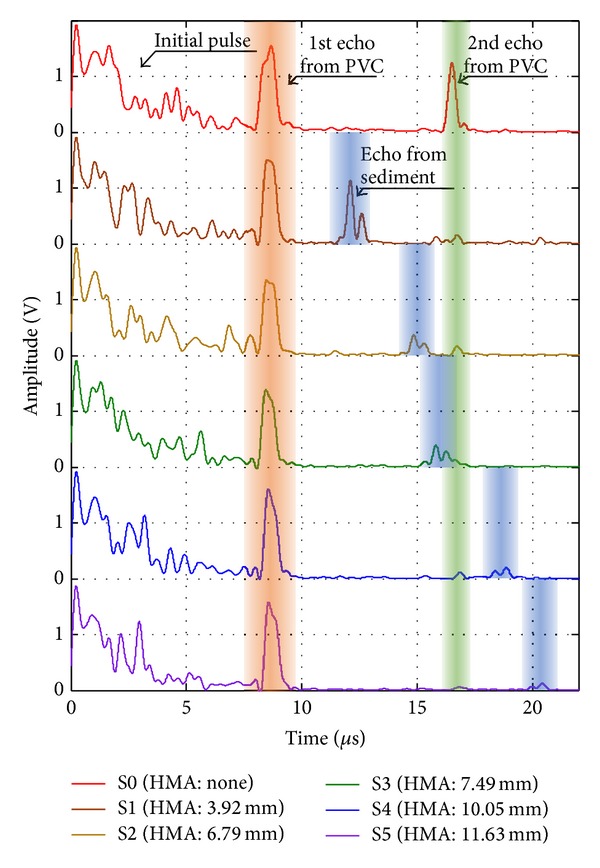
Absolute signal of the reflected ultrasonic wave.

In the experiment with the PVC pipe, likewise with obtaining the speed of sound in the PVC material, the speed of sound in the adhesive sediment was measured as about 2000 m/s. The signal processing with taking the absolute value and low pass filtering was applied to detect the pulse-echoes from the reflected signals measured from the PVC pipe as shown in Figure 9. The pulse-echoes from the wall of PVC pipe were detected at the specific time zones regardless of the thickness of the sediment that stuck on the wall. On other hand, the echo from the back surface of the sediment material was shifted depending on the thickness of them. The TOFs to the back surface of the sediment increased as shown in Table 3. The measured thickness of the sediment material was calculated from (3). The errors between actual and measured thicknesses were below 2.5%, which means that an ultrasonic pulse is able to measure the thickness of the substance that stuck on the wall of the PVC pipe.

## 5. Conclusions

The transmittance of ultrasonic wave in the PVC material and the applicability and effectiveness of the pulse-echo ultrasonic monitoring of the sediment which stuck on the inner wall of the PVC pipe were studied. For investigating the transmittance of acoustic energy in the PVC, the pulse-echo ultrasonic testing for the PVC sheets of different thickness was conducted. In addition, to simulate the sediment that stuck on the wall of the PVC pipe, the HMA was covered on the inner wall of the PVC pipe. The thicknesses of the sediment materials were measured through the pulse-echo ultrasonic testing and signal processing techniques.

In the experiment for PVC sheets, the speed of sound in PVC material was calculated as about 2258 m/s similar as the well-known speed of sound in PVC material. The echo signal was detected for the PVC sheet of 22 mm thickness. In the thickness measurement of PVC sheets with signal processing such as taking the absolute value and low pass filtering, the errors between actual and measured thicknesses of PVC sheets were below 5%. In the experiment for PVC pipe, the speed of sound in the hot adhesive material was calculated as about 2000 m/s. The errors between the actual and measured thicknesses of the stuck material were below 2.5%.

The results from the experiment showed the low errors between actual and measured thicknesses of the PVC material and the sediment. This supports that the pulse-echo ultrasonic inspection system is effective and applicable for the monitoring of sediments that stuck on the wall of the PVC pipe as well as the PVC material.

## Figures and Tables

**Table 1:** Time of flight and speed of sound.

Travel route	Initial to 1st peak	1st to 2nd peak	2nd to 3rd peak	3rd to 4th peak	Average
Time of flight (*μ*s)	2.600	2.607	2.733	2.695	**2.659**
Travel distance (mm)	6				**6**
Speed of sound (m/s)	2308	2302	2195	2226	**2258**

**Table 2:** The time of flight and the measured thickness of PVC sheets.

Specimen	T3	T5	T8	T10	T15	T22
Time of flight (*μ*s)	2.65	4.23	7.13	8.63	13.0	19.7
Actual thickness (mm)	3.0	5.0	8.0	10.0	15.0	22.0
Measured thickness (mm)	2.99	4.77	8.05	9.74	14.64	22.24
Error (%)	0.19	4.52	0.61	2.59	2.39	1.11

**Table 3:** The time of flight and the measured thickness of foreign substances.

Specimen	S1	S2	S3	S4	S5
Time of flight (*μ*s)	3.92	6.645	7.605	10.2	11.76
Actual thickness (mm)	3.92	6.79	7.49	10.05	11.63
Measured thickness (mm)	3.92	6.65	7.61	10.20	11.76
Error (%)	0.00	2.14	1.54	1.49	1.12
